# Digital health equity – A call to action for clinical and translational scientists

**DOI:** 10.1017/cts.2024.564

**Published:** 2024-10-09

**Authors:** Sheri Rowland, LaPrincess C. Brewer, Lisa G. Rosas

**Affiliations:** 1 University of Nebraska Medical Center College of Nursing, Lincoln, NE, USA; 2 Department of Cardiovascular Medicine, Division of Preventive Cardiology, Mayo Clinic College of Medicine, Rochester, MN, USA; 3 Department of Epidemiology and Population Health, Department of Medicine Division of Primary Care and Population Health, Stanford School of Medicine, Palo Alto, CA, USA

**Keywords:** Digital health equity, digital divide, under-represented minority populations, mHealth, digital health intervention

Digital health, such as mobile health (mHealth) technologies, wearable devices, and telemedicine, has enormous potential for improving prevention, diagnosis, and treatment of disease and increasing access to health information and healthcare. Digital health could potentially exacerbate health inequities given unequal access to technology and disparities in digital literacy across social groups [1]. For example, there is evidence from the COVID-19 pandemic that minoritized groups (e.g., Black, Latinx, and Indigenous patients), Non-English speakers, and those with low incomes had less access to telemedicine than non-Hispanic whites [2–5]. However, there is also a tremendous opportunity for Clinical and Translational Scientists to promote health equity with digital health by applying a health equity lens to the development, implementation, and evaluation of these approaches. Applying a health equity lens is a reflective process aimed at considering how digital health technologies may exacerbate health disparities focusing on those approaches that will lessen health disparities. The translational science community can play an important role in applying a health equity lens to digital health through education and training for clinical and translational scientists, the provision of resources, and policy change.

**Figure 1.**
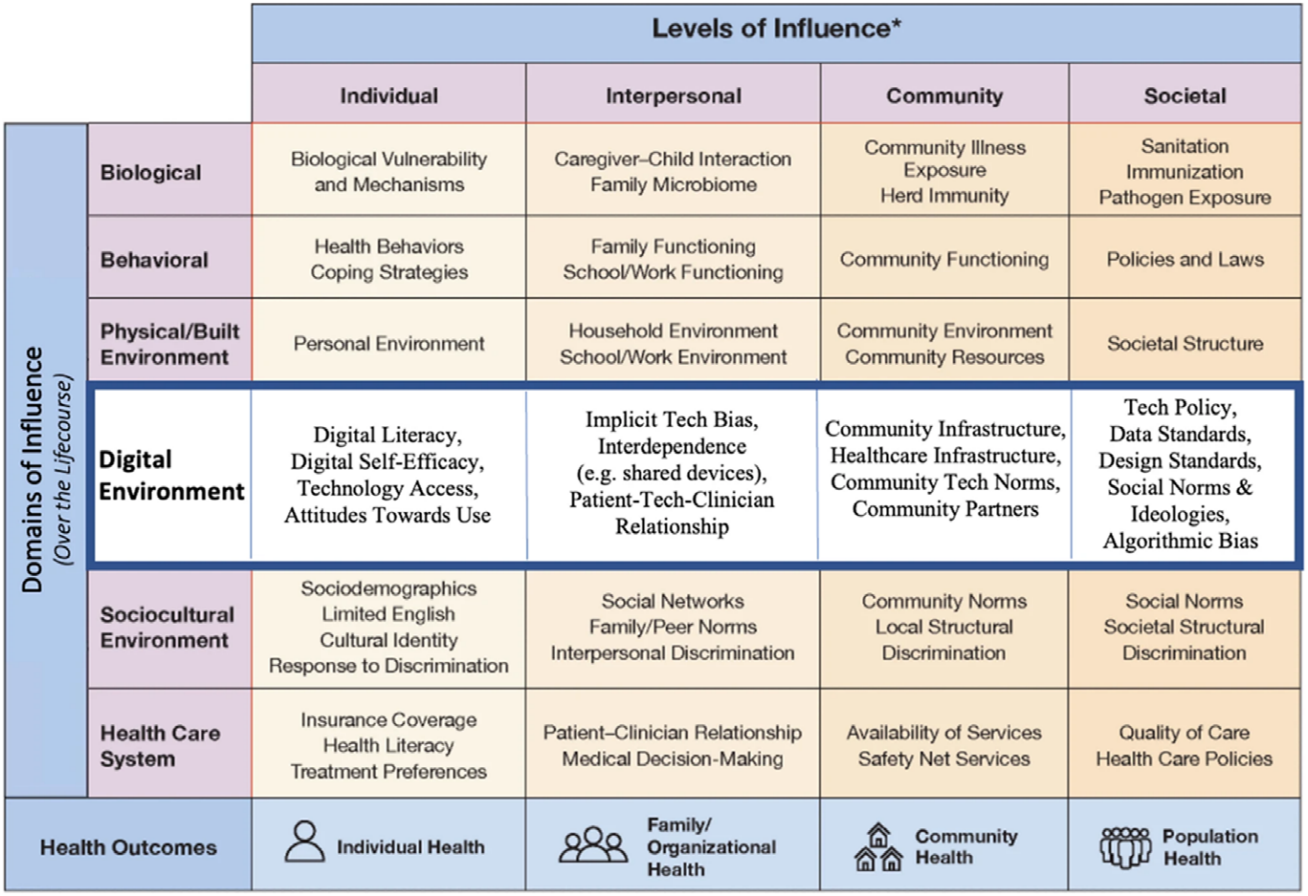
Framework for digital health equity.

**Figure 2.**
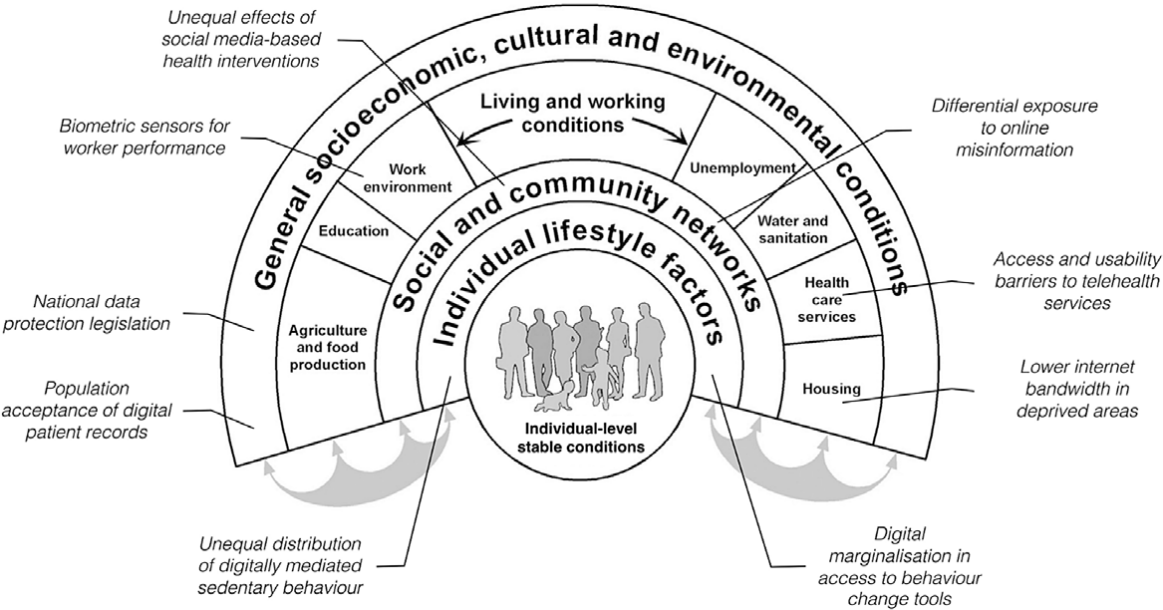
Digital Rainbow Model.

Education and training for clinical and translational scientists can leverage existing frameworks for digital health equity [6,7]. Two recently published frameworks are recommended for ensuring digital health interventions are inclusive, accessible, and beneficial: the Digital Health Equity Framework (Figure 1) [6] and the Digital Rainbow Model (Figure 2) [7]. The Digital Health Equity Framework builds on the National Institute on Minority Health and Health Disparities research framework [8] and emphasizes upstream approaches to scaling and implementing digital health interventions by focusing on factors impacting access and utilization such as broadband access, digital literacy, and cultural relevance. The Digital Rainbow Model has greater emphasis on social identity and participatory approaches [7]. Educational approaches (i.e., seminars, workshops, graduate course work, faculty trainings) grounded in these frameworks can guide researchers to consider structural, social, and environmental factors related to the digital divide in the development and implementation of new tools and technologies. For example, approaches guided by this framework could include providing hotspots to increase access to broadband for patients or digital navigators who can address barriers to technology access [9]. Professional organizations can provide training in theory-supported strategies for digital health equity in their specific domains. For example, the Society of Behavioral Medicine recently held a two-part webinar series: part I focused on design and implementation and part II focused on evaluation and dissemination. Key recommendations across both webinars centered on the idea that one size does not fit all with digital health interventions or programs. Community engagement, user-centered design, flexibility with technology options, digital health literacy, and resource allocation are essential elements of equitable digital health.

Applying a health equity lens to digital health requires resources that can be supported through Clinical and Translational Science Awards at Academic Medical Centers. Resources include those that support community and patient engagement in all stages of research including the development of digital tools, such as the Patient-Centered Outcomes Research Institute (PCORI) engagement rubric [10]. Engaging patients and communities with lived experience holds enormous potential for unlocking innovative approaches to promoting health equity, including those using digital health approaches. Resources for supporting patient and community engagement can include support in identifying appropriate partners, seed grants for developing and sustaining partnerships, and funding for engaging partners in developing and pilot-testing digital tools. Additionally, several national and international organizations have community-centered digital health guidelines including the Patient-Centered Outcomes Research Institute (PCORI) [11] and World Health Organization [12]. Another useful resource is the digital health checklist which guides health researchers to consider key digital health issues during project development including access, usability, privacy, data management, and risks/benefits [13]. Developed by digital health experts and grounded in ethical research principles, this checklist is now freely available online.

Lastly, Academic Medical Centers, funders, journals, and others can implement programs and policies that support digital health equity. For example, Academic Medical Centers, with the support of Clinical and Translational Science Awards, can provide programs that support and provide resources to researchers and their community/patient partners to address technology access and literacy issues at the outset of research projects. Publishers can motivate researchers to address digital health equity by requiring reporting on strategies used to address the digital health domains of influence. The journals *Annals of Behavioral Medicine, Translational Behavioral Medicine,* and all journals within the *Journal of the American Medical Association* network have recently implemented mandatory reporting on all categories of gender, race, ethnicity, and age for all research participants [14]. Presently, there are few reporting standards related to digital health intervention development (i.e., community engagement methods used); thus, more scientific bodies and professional organizations should mandate greater transparency [5,15].

Two recent community-based clinical trials highlight best practices through all stages of the research process when deploying digital health interventions in partnership with under-represented racial and ethnic minority populations. First, the FAITH! (Fostering African American Improvement in Total Health!) Trial was a cluster randomized controlled trial assessing the feasibility and preliminary efficacy of an mHealth intervention called the *FAITH! App* to promote cardiovascular health among African American adults (*N* = 85) [16]. This multi-funder-supported project (Clinical and Translational Science Awards program, American Heart Association, and NIMHD) transitioned an in-person cardiovascular health and wellness program into a smartphone app. Participatory design was used to co-translate the program with community members through an iterative process via focus groups. Potential end-user feedback enhanced the usability and satisfaction prior to rigorously testing the app’s impact on cardiovascular health. Guided by principles of digital health social justice [17], the app development process accounted for issues of accessibility, data security, and community technology norms. The second example of applied health equity best practices when conducting digital health interventions is the Hombre Trial [18]. This PCORI-funded randomized controlled study tested a culturally adapted intervention to promote weight loss in Latino men with overweight or obesity (*N* = 424). The intervention allowed the men to self-select on how to receive the coach-facilitated group sessions intervention: in-person, through videoconferencing, or viewing pre-recorded group sessions [19]. Allowing the men to choose the delivery mode was key to engaging this historically marginalized population and was only possible through formative work pilot-testing of the video conferencing. Both exemplar projects integrated advisory boards comprised of community and patient stakeholders who, through regular contact with the research team, advised on cultural adaptations of the interventions through semi-structured interviews, discussion forums, and focus groups. In both examples, flexibility with design and approach was key to recruiting and retaining participants. Notably, both case examples demonstrated clinically significant changes in health outcomes with at-risk populations who are largely under-represented in clinical and translational research.

In conclusion, digital health technologies offer great potential for improving healthcare access, delivery, and outcomes. Clinical and translational scientists are called to apply a health equity lens to the development and evaluation of digital health technologies to avoid exacerbating current health inequities and to increase the potential that these approaches will contribute to promoting health equity. This includes seeking out educational opportunities in digital health equity, especially those that use evidence-based frameworks [2,3], and engaging those with lived experience in all phases of the research process to develop and evaluate digital health technologies. It is especially important to share methods and findings from engaging those with lived experience in the research process on digital health so that others can learn from and build on this knowledge. Finally, we also call on Academic Medical Centers, funders, and professional societies to provide educational opportunities and resources for applying an equity lens to the field of digital health.
